# Dental Ethics

**Published:** 1891-11

**Authors:** W. A. Knowles


### Dental Ethics.

BY W. A. KNOWLES, M.D., D.D.S.

Read before the California State Dental Association.

*Mr. President and Gentlemen:—*

In the busy whirl of the competitive system those engaged in business are often less solicitous for the welfare of others than for their own individual success.

The very nature of competition is such that it seems impossible for one to succeed except it be at the expense of others; that in climing the ladder of success it is held to be necessary that some of its rounds should be formed by the bodies of those who have been unsuccessful.

Competition is so great that all manner of methods must be adopted to direct attention towards those who are endeavoring to succeed.

Competition frequently causes the cost to the consumer to be lower, but does not necessarily imply that such articles will be *cheaper*.

Competition reduces the profits to the dealer, and not infrequently the *quality* to the consumer, and it would be a pertinent question to ask in such cases as to who is benefited.

The statement has been made that it is impossible for one to be strictly honest in business and succeed. The truth of this statement must be for each to decide individually, but the fact will be acknowledged that competition does not cultivate the most desirable traits of the human character.

While advertising and other "business" methods may be tolerated in a business it is altogether a different matter in the humanitarian professions of medicine and dentistry. Those professions which have for their object the alleviation of pain and suffering should be above the mere love of money-making.

It is impossible to extend the sphere of a humanitarian profession by the employment of additional persons as is done in business; individual and personal service being expected and demanded by patients.

A profession should not, consequently, be expected to yield such a harvest as a successful business would, and he makes a mistake who enters a humanitarian profession for the sole purpose of gaining riches.

What are we to think of the professional man who said that when he was called to a case he first ascertained the condition of his patient's pocket? Should not such a one be called by his appropriate name, a highway robber?

The true professional instinct causes one to act from the love of his calling, and makes the fee for the service rendered, a matter of secondary consideration.

The curse of professions has been that many individuals have entered them for the sake of the fees, or because such professions seem to them an easy way of obtaining a good living.

Such persons, in order to succeed, introduce methods such as are to be considered legitimate in *business*, and they endeavor to conduct their professional affairs in a similar manner.

If the result of such methods in business is doubtful, what will it be in professional affairs?

The tendency is to drag the profession down to the level of a competitive business, and here it is that the consumer or recipient of service is the sufferer, for the temptation would be to charge the highest possible fee, and to use the shortest amount of time in performing the service.

Professions have been brought to their present standards of excellence by the unselfish generosity of men who have preceded us, and now, as in times gone by, there are to be found upon the outskirts of the profession, and even at times pressing forward into the main body, those parasites who endeavor to drag the professions down to the dust.

Let us remember that whatever standing the professions now have is largely the result of the efforts of those who have gone before us, but upon us depends whether the standard shall rise or fall. Let us, therefore, do all in our power to elevate the professions, and ourselves refrain from, and discourage in others, every thing having tendency to lower the standards.

As long as fees are large enough to tempt the cupidity of money-loving individuals, so long will those persons having no loftier ambition enter into professional ranks.

To this class of practitioners the human body is but the ground wherein to delve for dollars. The dollars seem to be the main consideration.

They seem to change the old saying to "Physician, *heal* thyself."

For the sake of the legitimate practitioners in professions the highest standards should be maintained, and in order to do so it is necessary to encourage all true professional and humanitarian instincts, and to frown down any and every thing which has the least tendency to detract from, or drag down the professions from the high standards which they are endeavoring to maintain.

Charlatanry, like gravitation, if allowed to operate unchecked will drag every thing down to one common level.

Of late years there seems to be a tendency, in some instances, to overstep some of the boundaries which codes of ethics place about professional associations.

Whether it is due to fields of labor becoming more limited as the ranks of the profession fill up, or to any other cause, if persisted in the result can not help but be to contaminate and degrade.

No profession is safe if men of prominence will permit or countenance breaches of ethical codes, because the successful practitioners are those to whom all others look for guidance and example, and if these rods turn out to be but reeds, what must the effect of such pernicious examples be upon the remainder of the professions?

To those who have hoped to see the profession of dentistry raised to its proper level it is humiliating to see the ignorance of many practitioners.

What would be thought of medical practitioners who would circulate, recommend or use nostrums, patented or secret preparations upon their patients? And yet how many dentists are guilty of doing this very thing?

How can the dental profession expect general recognition when it will countenance and do such things?

Has it not come to a pretty pass when the most eminent men in our profession will endorse and recommend a secret preparation for alleviating pain, in and about the teeth, when upon analysis such preparation is found to contain arsenic in large and poisonous quantity—where as much as ten grains of arsenic are put into a person's hands without, absolutely, no caution as to its use, and furthermore, under the statement that it is perfectly harmless?

In the first place, what happens to the nerves of teeth to which this application is made?

How can men recommend and sanction the use of their names in connection with preparations of which they do not know the ingredients?

Members of associations which are governed by the Code of Ethics wherein it is distinctly stated that it is unprofessional to recommend or circulate nostrums, will purchase every secret preparation which is put before them if it gives any promise of rendering the operation of extracting less painful.

Suppose a secret preparation to be injected hypodermically, and suppose bad results follow.

How can the dentist, or the physician he may summon, know what to do when the nature of the injection causing the trouble is not known?

Our professions are governed by codes of ethics both written and unwritten. The written portions are generally observed by reputable practitioners, but the unwritten portions are not always so well lived up to.

The remedy, therefore, must be to particularize more and to transfer many portions of the unwritten to the written code, thus drawing the lines more strictly.

Some persons have queer ideas in regard to professional matters.

A certain dental journal instituted the practice of propounding a question each month in regard to dental subjects and requested answers from its readers.

A question in one issue was as to what operation would be best in case the crown of a central incisor should be lost, and the root remain intact in the mouth of a ten year-old-child?

Most of the answers recommended the insertion of a pivot tooth of one of the many good patterns, but one practitioner answered that he would build it up with gold and it would be a fine advertisement for him. Think of the professional aspirations of such a one and how he would, with the basest of motives, prostitute the profession which he represents. Let us ever bear in mind that the mantle of professional standing is woven from the threads of our individual actions.

The strict observance of all teachings of conscience may not always fill the purse so rapidly, but it makes the mind clearer, the heart happier, and the meeting with death, joyous.

